# Antimicrobial Resistance Profiles for Different Isolates in Aden, Yemen: A Cross-Sectional Study in a Resource-Poor Setting

**DOI:** 10.1155/2020/1810290

**Published:** 2020-04-22

**Authors:** Wafa F. S. Badulla, Mohammed Alshakka, Mohamed Izham Mohamed Ibrahim

**Affiliations:** ^1^Department of Analytical Chemistry, Faculty of Pharmacy, Aden University, Aden, Yemen; ^2^Section of Clinical Pharmacy, Faculty of Pharmacy, Aden University, Aden, Yemen; ^3^Department of Clinical Pharmacy and Practice, College of Pharmacy, QU Health, Qatar University, Doha, Qatar

## Abstract

**Background:**

There is a rapid deterioration in the effectiveness of antibiotics due to the global prevalence of bacterial antimicrobial resistance (AMR). AMR can cause an increase in mortality and morbidity due to treatment failures and a lack of effective therapy.

**Objective:**

The purpose of this study was to evaluate the AMR pattern of different bacterial isolates at hospitals and laboratories.

**Materials and Methods:**

A cross-sectional study from March 2019 to June 2019 was conducted at different governmental and private hospitals and laboratories in Aden, Yemen. Age, sex, specimen type, bacterial isolates, and antibiotic susceptibility pattern were collected using a data extraction sheet. Descriptive statistics were used for data analysis.

**Result:**

Data were recorded for 412 patients from whom 20 clinical specimens were collected and analyzed. The most common bacteria isolated were *Staphylococcus* spp. (*n* = 172, 41.74%), *E. coli* (*n* = 164, 39.80%), *Pseudomonas* spp. (*n* = 37, 8.98%), and *Klebsiella pneumoniae* (*n* = 18, 4.36%); other bacteria were less common. The overall bacterial resistance was highest against the combination of sulfamethoxazole with trimethoprim (73.12%), followed by amoxicillin and clavulanate (65.19%). The cephalosporin antibiotics also showed high resistance rates. The study also showed moderate bacterial resistance to gentamycin (32.65%), azithromycin (29.92%), cefoxitin (62.65%), and ciprofloxacin (25.60%). Ertapenem (16.67%) and levofloxacin (15.56%) had the lowest resistance rates.

**Conclusion:**

There was a high percentage of bacteria resistant to several antibiotics. Antibiotic susceptibility testing is a prerequisite guide for the selection of appropriate antibiotic therapy for bacterial infections.

## 1. Introduction

The problem of widespread resistant bacteria has become a major threat to reduce the effectiveness of antibiotics worldwide [1–3]. The emergence of multiresistant organisms is escalating, especially in the developing world [4]. As stated by the WHO, there is a global need to increase awareness about antimicrobial resistance (AMR) [5–7]. This awareness and monitoring of AMR will be reflected in several aspects involving reporting the bacterial resistance rate, a guide for the selection of suitable antibiotics, and reducing hospitalization frequency, treatment costs, and the death rate [8–10].

To obtain information about the AMR in a country, there should be continuous surveillance that involves the collection of antimicrobial susceptibility testing (AST) results carried out by microbiology laboratories on clinical specimens. The obtained data provide insight into the prevalence of antibiotic-resistant bacteria in that country and help in decision-making, mainly the selection of antibiotics to be applied either for empirical treatment of sick patients or prophylaxis in patients at enhanced risk of infection. The national treatment guidelines should be updated according to the new data.

In Yemen, due to low income, most physicians try to reduce the cost of laboratory tests by treating patients according to previous clinical experience (i.e., empirical therapy) without the use of diagnostic testing, which should be performed by laboratory professionals. The situation may have been effective before the wide spread of resistant microorganisms. However, due to the serious risks of multiresistant organisms, a guide for the selection of appropriate antibiotics for patients' illnesses should be provided by the assistance of an effective laboratory system instead of depending on empirical therapy (irrational use of antibiotics). Information on infection epidemiology and antimicrobial resistance patterns is a critical means to help physicians empirically treat life-threatening infectious illnesses. Several studies reported a correlation between increasing the AMR rate and increasing antibiotic use [4, 11–14].

In a study performed in 2015 to explore the antibiotic-prescribing manner of physicians in outpatient sectors of hospitals in Aden, Yemen, the number of prescriptions containing antibiotics was 84.2%, which is far from the standard values suggested by the WHO [15].

Another problem that faces Yemen is counterfeit medicines, and the smuggling of medicine is widespread as well. According to a study carried out in Yemen, approximately 80% of medicines entered the country via illegal routes, and approximately 40% were fake or of low quality [16]. According to the WHO, approximately 43% of falsified antibiotics have no active ingredients, 24% are of bad quality, 21% have a reduced quantity of an active ingredient, and 7% have wrong ingredients [17]. The quality control of antibiotics is a very significant issue because counterfeit-quality generic antibiotics may contribute to the emergence of resistant bacteria [18].

The main aim of the current study was to determine the different AMR patterns of bacterial isolates from different specimens at various government and private hospitals as well as diagnostic service laboratories in Aden, Yemen. Findings from this study will guide the rational use of the existing antimicrobials and guide optimal empiric treatment in critically ill patients.

## 2. Materials and Methods

### 2.1. Study Design

A cross-sectional study was carried out from March 2019 to June 2019. The study was conducted at different governmental and private hospitals and laboratories in Aden, Yemen: National Blood Transfusion and Research Center, Al-Medina Medical Laboratories, Babel Hospital, and Aden German International Hospital. The patient data were collected from the microbiology laboratory unit registration book. Age, sex, specimen type, bacterial isolates, and antibiotic susceptibility pattern were collected using a data extraction sheet.

### 2.2. Isolation and Identification of Bacteria

All clinical samples were collected and identified for pathogenic bacteria by standard microbiological techniques. According to the source of clinical specimens, each sample was placed onto the specific agar for each bacterium (HiMedia Laboratories Pvt. Limited, India) and then incubated aerobically at 37°C for 24 h. Standard microbiological methods were applied for the identification of the bacterial species [19].

### 2.3. Antimicrobial Susceptibility Testing

Antimicrobial susceptibility tests were carried out by all laboratories using the disk diffusion method [20].

### 2.4. Quality Control

A standard bacteriological technique was applied to maintain accurate laboratory test results. American Type Culture Collection (ATCC) standard reference strains *Escherichia coli* (*E. coli*) ATCC-25922, *Staphylococcus aureus* (*S. aureus*) ATCC 25923, and *Pseudomonas aeruginosa* (*P. aeruginosa*) ATCC 25853 were used to control the quality of culture and drug susceptibility testing.

### 2.5. Data Analysis

Statistical Package for Social Sciences (SPSS) version 21 (IBM Corp. released 2012; IBM SPSS Statistics for Windows, version 21.0, Armonk, NY: IBM Corp.) was used for both data entry and analysis. Descriptive statistics were used to determine the frequency of isolated bacteria. The antibiotic susceptibility to the used antibiotics was analyzed.

### 2.6. Ethical Consideration

The study protocol was approved by the Ethics Research Committee of the Faculty of Medicine and Health Sciences, Aden University (research code: REC-61-2019).

## 3. Results and Discussion

### 3.1. Study Population and Laboratory Distribution

A total of 412 recorded patient data points were collected from four laboratories. A majority of samples (234, 56.8%) were collected from female patients, and 178 (43.2%) were collected from male patients. A higher isolation rate was obtained from adults than from children (*n* = 355, 86.2%) (see Table 1).

### 3.2. Bacterial Isolates

The distribution of bacterial isolates with the associated specimens is illustrated in Table 2. In this study, a total of 20 clinical specimens were collected from various clinical specimens. The most common specimens processed in the different laboratories were urine (*n* = 150, 36.4%), followed by pus swabs (*n* = 69, 16.7%) and wound swabs (*n* = 50, 12.1%). The majority of the bacterial isolates were recovered from the urine specimens. The most common bacteria isolated were *Staphylococcus* spp. (*n* = 172, 41.74%), *E. coli* (*n* = 164, 39.80%), *Pseudomonas spp.* (*n* = 37, 8.98%), and *Klebsiella pneumoniae* (*n* = 18, 4.36%).

### 3.3. Antibiotic Nonsusceptibility

A total of 63 antibiotics were screened by AST. The most frequently tested antibiotics were studied for their AMR (Table 3). The table summarizes the bacterial resistance patterns for the selected antibiotics. The overall resistance rate was highest in the case of the sulfamethoxazole and trimethoprim combination, at 73.12%, while the lowest resistance rate was observed for ertapenem (carbapenem antibiotic), at 16.67%.

AMR in Yemen can cause an increase in mortality and morbidity due to treatment failures and lack of effective therapy. Moreover, in a country with poor resources, such as Yemen, the economic consequences may be even more detrimental, as increased treatment costs consume funds. The development of highly resistant pathogens is the inevitable outcome for the frequent, irrational use of broad-spectrum antibiotics as well as the availability of antibiotics as over-the-counter medicines in this city. The present study provides an outline of the existing resistance level that can be used as a guide for effective treatment. The data were collected from four main hospitals and laboratories in this city. Each laboratory throughout the study period reported variable numbers of antimicrobial-resistant bacterial strains from approximately 20 different kinds of medical specimens. The bacterial isolate distribution and frequency varied in different clinical specimens. The number of male patients was higher than that of female patients, and the number of adults was higher than that of children.

The findings from the current study revealed the distribution of bacterial isolates with the specimens and the AMR patterns for different pathogenic isolates. The key outcome is the prevalence of *Staphylococcus* spp. in the main specimens (urine, wound, ear, nasal, vaginal swab, etc.), at 41.74%. This finding is similar to that in a study carried out in Gabon and Tanzania [21–23]. Additionally, *E. coli* and *Pseudomonas* spp. were dominant in the isolates. These species are commensal bacteria that can be present as normal flora on different parts of healthy individuals. However, these bacteria can be pathogenic and readily disseminated. The high variety of pathogens isolated from different specimens indicated the ineffectiveness of using empirical antibiotic therapy and supported the use of susceptibility tests before prescribing antibiotic therapy. However, these data can be a guide for empirical antibiotic therapy for the most predominant pathogens for different specimens.

The findings show the bacterial resistance patterns to the selected antibiotics. The highest overall resistance was for the combination of sulfamethoxazole with trimethoprim (cotrimoxazole), followed by amoxicillin and clavulanate, which could be due to recurrent empirical therapy with these combinations prior to sampling. The majority of bacteria resistant to sulfamethoxazole and trimethoprim were gram-negative species, namely, *E. coli*, *Klebsiella* spp., and *Pseudomonas* spp., while in the case of amoxicillin and clavulanate, most bacteria (gram negative and positive) had a high percent of resistance. The result for cotrimoxazole is identical to that in a study in Ghana, but it is dissimilar for amoxicillin and clavulanate; the study revealed a low level of resistance to amoxicillin and clavulanate [24].

In addition, some of the cephalosporin antibiotics had high resistance percentages, such as cefepime 64.60%, ceftriaxone 62.20%, cefuroxime 54.36%, and ceftazidime 47.54%. This finding is an indication of the irrational use of new and strong antibiotics for simple infectious or even viral diseases that leads to widespread bacterial resistance to these antibiotics. This result is aligned with 2014 WHO reports [25]. Since 2004, resistance to cephalosporins has increased seriously for *Escherichia coli* infections [26]. In addition, parallel findings were obtained from studies carried out in Iraq, Pakistan, and Ethiopia [27–30].

This study also showed moderate bacterial resistance toward gentamycin, azithromycin, cefoxitin, and ciprofloxacin. *Klebsiella* spp. showed the highest resistance to gentamycin (62.50%), followed by *E. coli* (39.13%). The resistance was lower than that in studies carried out in India, where the resistance of *Klebsiella pneumoniae* to gentamycin reached 80% in one study and 100% in another study. In the case of *E. coli*, the resistance ranged from 48.20% to 92.4% [31, 32]. The WHO GASP statistics of several countries from 2009 to 2014 presented continual prevalent *gonococcal* resistance to azithromycin, but azithromycin was still effective against *Shigella* sp. strains [33]. Cefoxitin is a second-generation cephalosporin and exhibited the lowest resistance rate among the cephalosporin generations tested in the current study.

Ciprofloxacin is used as a first-choice treatment for several infections, such as UTI, GIT infection, and typhoid, in Yemen. The widespread use of these antimicrobials in the treatment of community-acquired infections may have contributed to the high levels of observed resistance. The percentages of ciprofloxacin resistance were variable according to the site of bacterial infection as well as the geographical site. The resistance percentages are high in developing countries in comparison to those in developed countries. *Klebsiella* spp. showed the highest resistance rate, 41.66%. A study carried out in Iran showed a 38.8% resistance rate of *Klebsiella pneumoniae* to ciprofloxacin [34] and approximately 28.5% in community-acquired UTI [35]. Another study conducted in India revealed that the resistance rate of *Klebsiella pneumoniae* to ciprofloxacin was 80% [36]. The current study showed that the resistance rate was 41.66%, which is close to that in the study from Iran and lower than that in the study from India.

Levofloxacin and ertapenem had the lowest resistance rates. Several studies revealed increased resistance due to the frequent utilization of levofloxacin [37–39]. The low bacterial resistance to ertapenem might be due to its relatively new administration and low rate of prescription for infectious illness. Studies in Ethiopia and Colombia also reported low resistance to ertapenem [40, 41].

*Enterococci* spp. were detected in limited numbers and were not susceptible to antibiotics such as cefotaxime (*n* = 1/1, 100%) and gentamycin (*n* = 1/2, 50%). A study carried out in Iran on the occurrence of *Enterococcus faecalis* and *Enterococcus faecium* in several clinical samples indicated a nonsusceptible pattern toward rifampicin (*n* = 122, 76.25%) as well as erythromycin (*n* = 117, 73.12%). The same study also showed the prevalence of virulent traits [42]. Another review study in Iran (2009 to 2015) revealed the emergence of *Acinetobacter baumannii* clinical specimens with high resistance to different classes of antibiotics (including carbapenems) [42]. The result of this study was in parallel with that of a study carried out in Iran (2015-2016) [43]. Multidrug-resistant *Acinetobacter baumannii* strains have become a worldwide problem [44]. Although this type of bacterium was not detected in any of the current study clinical specimens, it is possible that this type of bacterium would also show multidrug resistance if it was examined. *Enterobacter* spp. were resistant to amoxicillin with clavulanate (*n* = 1/1, 100%), cefepime (*n* = 1/1, 100%), cefotaxime (*n* = 3/3, 100%), ceftazidime (*n* = 1/1, 100%), cefuroxime (*n* = 1/1, 100%), gentamycin (*n* = 1/2, 50%), and ciprofloxacin (*n* = 1/5, 20%). The results revealed that bacteria were highly resistant to the cephalosporin, with moderate resistance against gentamycin and relatively low resistance to ciprofloxacin. The results of a study in Iran on an *Enterobacter cowanii* isolate from powdered infant formula revealed susceptibility to imipenem, meropenem, ceftazidime, ciprofloxacin, and colistin. Fifty percent of isolates were resistant to ampicillin, amoxicillin, and cotrimoxazole [45].

It is worth mentioning that many bacteria had multidrug resistance to the most examined antibiotics. This finding corresponds with studies in Ethiopia [33, 46, 47]. It is an expected outcome due to the worldwide existence and prevalence of multidrug-resistant clones [48–50]. Similarly, a study in Iranian hospitalized patients with cancer showed significant spreading of MDR gram-negative bacteria, which may raise the problem of healthcare-associated infections in cancer patients [51, 52].

Initially, antibiotic-resistant bacteria were limited to hospitals; nevertheless, resistant strains of bacteria are currently present everywhere. It is expected that by 2050, none of the currently present antibiotics will be effective in treating infections. The situation will be tragic unless a new drug is developed or discovered [53].

Nevertheless, the prescription patterns should be altered to keep pace with the changes in bacterial resistance.

The comparison to the other studies is limited to the most similar due to the variance in the pattern and frequency of bacterial isolates among different clinical specimens in the present literature, which might be due to differences in study objectives, study designs, qualification methods, geographic dissimilarities, and discrepancies in the study populations. This report is considered the tip of the iceberg for the situation of bacterial resistance in Aden city. These results can serve as a basis for upcoming prospective and retrospective studies to have a clear idea about the change in bacterial resistance patterns over the years. In Yemen, antibiotics are important and often scarce resources. They are commonly unregulated, misused, and overused. There is a need to increase access to diagnostic laboratories, enhance surveillance of the emergence of AMR, and conduct additional studies.

This study has a few limitations. First, the small sample size, which may not characterize the overall population, lived in the study area. Second, the sources of pathogens were indistinct; they were either community- or hospital-acquired infections. Furthermore, we could not obtain the history and state of the diseases if the patient used antibiotics before the susceptibility test or not. Epidemiological data (e.g., place of residence and the onset of disease) have not been reported.

Few recommendations can be considered based on the study findings. The outcome is worrisome and requires an extended study that involves all Yemeni cities to obtain a clear view of antimicrobial resistance. The effort of gathering information about the trends of bacterial resistance will be unprofitable if there are no local, national, and global strategies to provide effective interventions to reduce the prevalence of AMR, especially in a resource-poor setting. The following points should be taken into consideration: (1) a study with a larger sample size with more demographic, epidemiological, and health information of each patient should be conducted; (2) a retrospective study for the past 10 years is recommended to compare the bacterial resistance patterns; (3) there should be continuous surveillance of antibacterial resistance with regular reporting of data to the WHO and participation in the Global Antimicrobial Resistance Surveillance System (GLASS); and (4) diagnostic stewardship should be activated to provide appropriate interventions and application of suitable antimicrobial agents for the patient's infections.

## 4. Conclusions

The information within this study provides essential data on antimicrobial resistance in Aden, Yemen. This study revealed that *Staphylococcus* spp., followed by *E. coli*, *Pseudomonas spp.*, and *Klebsiella pneumoniae*, were the most widespread pathogenic bacteria in several isolates. Overall bacterial resistance was common for old antibiotics, such as the combination of sulfamethoxazole with trimethoprim, followed by amoxicillin and clavulanate. Additionally, cephalosporin had a relatively higher resistance rate than other antibiotics. The study also showed moderate bacterial resistance toward gentamycin, azithromycin, cefoxitin, and ciprofloxacin. However, a lower percentage of resistance was present for the combination of ampicillin with sulbactam, ertapenem, and levofloxacin. Most isolated bacteria showed multidrug resistance. Consequently, antibiotic susceptibility testing is an important guide for the choice of suitable antibiotic treatment for bacterial infections.

## Figures and Tables

**Table 1:** Sex, patient category, and laboratory distribution.

	Frequency	Percentage
Gender		
Male	178	43.2
Female	234	56.8
Patient category		
Child	57	13.8
Adult	355	86.2
Study location		
National Blood Transfusion and Research Center	35	8.5
Al-Medina medical laboratories	234	56.8
Babel Hospital	41	10.0
Aden German International Hospital	102	24.8

**Table 2:** Distribution of bacterial isolates with the specimens.

Specimen	Bacteria	*n* (%)	Total
Urine	*E. coli*	90 (60%)	150
	*Staphylococcus* species	38 (25.33%)	
	*Pseudomonas* species	13 (8.66%)	
	*Enterobacter* species	2 (1.33%)	
	*Enterococci* species	1 (0.66%)	
	*Klebsiella pneumoniae*	2 (1.33%)	
	*Candida albicans*	2 (1.33%)	
	*Streptococcus* species	1 (0.66%)	
	*Sphingomonas paucimobilis*	1 (0.6%)	

Plural	*Staphylococcus* species	3 (50%)	6
	*Salmonella Paratyphi A*	2 (33.33%)	
	*E. coli*	1 (16.67%)	

Ear swab	*Staphylococcus* species	16 (72.72%)	22
	*Pseudomonas* species	3 (13.63%)	
	*Proteus* species	2 (9.09%)	
	*E. coli*	1 (4.54%)	

Wound swab	*Staphylococcus* species	25 (49.01%)	51
	*E. coli*	14 (27.45%)	
	*Pseudomonas* species	4 (7.84%)	
	*Proteus* species	3 (5.88%)	
	*Streptococcus* species	2 (3.92%)	
	*Klebsiella pneumoniae*	2 (3.92%)	
	*Enterobacter* species	1 (1.96%)	

Throat swab	*Staphylococcus* species	2 (40%)	5
	*Streptococcus* species	2 (40%)	
	*Klebsiella pneumoniae*	1 (20%)	

Vaginal swab	*Staphylococcus* species	16 (42.10%)	38
	*E. coli*	15 (39.47%)	
	*Klebsiella pneumoniae*	3 (7.89%)	
	*Pseudomonas* species	3 (7.89%)	
	*Candida albicans*	1 (2.63)	

Nasal swab	*Staphylococcus* species	3 (75%)	4
	*E. coli*	1 (25%)	

Sputum	*E. coli*	3 (37.5%)	8
	*Pseudomonas* species	2 (25.00%)	
	*Klebsiella pneumoniae*	1 (12.50%)	
	*Enterobacter* species	1 (12.50%)	
	*Candida albicans*	1 (12.50%)	

Blood	*Staphylococcus* species	13 (92.85%)	14
	*Pseudomonas* species	1 (7.14%)	

Cervical secretion	*Staphylococcus* species	2 (66.66%)	3
	*E. coli*	1 (33.33%)	

Skin specimen	*Pseudomonas* species	3 (100%)	3

Pus swab	*Staphylococcus* species	41 (59.42%)	69
	*E. coli*	20 (28.98%)	
	*Klebsiella pneumonia*	4 (5.79%)	
	*Pseudomonas* species	4 (5.79%)	

Nasopharynges	*Staphylococcus* species	4 (100%)	4

Semen	*Staphylococcus* species	6 (66.66%)	9
	*Klebsiella pneumoniae*	2 (22.22%)	
	*Pseudomonas* species	1 (11.11%)	

Cerebrospinal fluid	*Pseudomonas* species	3 (75%)	4
	*E. coli*	1 (25%)	

Stool	*E. coli*	16 (88.88%)	18
	*Staphylococcus* species	2 (11.11%)	

Catheter swab	*Candida albicans*	1 (100%)	1

Eye swab	*Staphylococcus* species	1 (100%)	1

Tracheostomy swab	*Pseudomonas* species	1 (100%)	1

Rectal swab	*E. coli*	1 (100%)	1

**Table 3:** Bacterial resistance patterns to the selected antibiotics.

Type of isolate	Antimicrobial nonsusceptibility						
	Amoxicillin with clavulanate *N*/total (%)	Ampicillin with sulbactam *N*/total (%)	Azithromycin *N*/total (%)	Cefepime *N*/total (%)	Cefotaxime *N*/total (%)	Cefoxitin *N*/total (%)	Ceftriaxone *N*/total (%)
*Klebsiella* species	5/8 (62.50)	0/1(0)	2/6 (33.33)	5/7 (71.43)	2/6 (33.33)	1/4 (25.00)	0/3 (0)
*E. coli*	71/98 (72.40)	8/20 (40.00)	7/23 (30.43)	61/89 (68.54)	76/111 (68.47)	23/85 (27.05)	77/114 (32.45)
*Pseudomonas* species	24/27 (88.88)	4/5 (80.00)	1/9 (11.11)	14/24 (58.33)	7/7 (100)	6/8 (75.00)	9/13 (69.23)
*Staphylococcus aureus*	10/16 (62.50)	1/31 (3.22)	27/85 (31.76)	20/33 (60.60)	14/29 (48.27)	12/65 (18.46)	17/33 (51.51)
*Streptococcus* species	6/29 (20.68)	1/31 (3.22)	0/1 (0)	2/3 (66.66)	1/3 (33.33)	ND	1/2 (50.00)
*Proteus* species	1/2 (50.00)	ND	1/2 (50.00)	0/3 (0)	0/4 (0)	1/2 (50.00)	0/2 (0)
*Enterobacter* species	1/1 (100)	ND	0/1 (0)	1/1 (100)	3/3 (100)	0/1 (0)	ND
*Enterococcus* species	ND	ND	ND	ND	1/1 (100)	ND	ND
*Salmonella Paratyphi A*	ND	ND	ND	ND	1/1 (100)	ND	2/3 (66.66)
*Sphingomonas paucimobilis*	ND	ND	ND	1/1 (100)	0/1 (0)	1/1 (100)	ND
Total	118/181 (65.19)	21/97 (21.65)	38/127 (29.92)	104/161 (64.60)	104/166 (62.65)	44/166 (26.50)	107/172 (62.20)

Type of isolate	Antimicrobial nonsusceptibility						
	Ciprofloxacin *N*/total (%)	Ceftazidime *N*/total (%)	Cefuroxime *N*/Total (%)	Ertapenem *N*/total (%)	Gentamycin *N*/total (%)	Levofloxacin *N*/total (%)	Sulfamethoxazole with trimethoprim *N*/total (%)
*Klebsiella* species	5/12 (41.66)	1/3 (33.33)	4/5 (80.0)	1/5 (20.0)	5/8 (62.50)	0/1 (0)	2/3 (66.66)
*E. coli*	28/103 (27.18)	12/29 (41.38)	19/26 (73.07)	6/89 (93.25)	9/23 (39.13)	11/40 (27.5)	57/75 (76.0)
*Pseudomonas* species	10/28 (35.71)	8/17 (47.05)	14/19 (73.68)	3/4 (74.0)	5/16 (31.25)	0/13 (0)	9/15 (60.0)
*Staphylococcus aureus*	19/95 (20.0)	7/11 (63.63)	15/45 (33.33)	9/17 (52.94)	12/45 (26.66)	9/73 (12.32)	49/66 (74.24)
*Streptococcus* species	ND	ND	1/2 (50.0)	1/2 (50.0)	0/1 (0)	ND	0/1 (0)
*Proteus* species	0/1 (0)	ND	1/2 (50.0)	0/2 (0)	0/1 (0)	0/2 (0)	ND
*Enterobacter* species	1/5 (20.0)	1/1 (100)	1/1 (100)	0/1 (0)	0/1 (0)	1/2 (50.0)	ND
*Enterococci* species	ND	ND	0/1 (0)	ND	1/2 (50.0)	0/2 (0)	ND
*Salmonella Paratyphi A*	0/1 (0)	ND	1/2 (50.0)	ND	ND	0/2 (0)	ND
*Sphingomonas paucimobilis*	0/1	ND	ND	ND	0/1 (0)	ND	ND
Total	63/246 (25.60)	29/61 (47.54)	56/103 (54.36)	20/120 (16.67)	32/98 (32.65)	21/135 (15.56)	117/160 (73.13)

## Data Availability

Data can be obtained from the authors upon request.
